# Mediating social support through sensor-based technologies for children’s health behavior change

**DOI:** 10.1093/jcmc/zmad011

**Published:** 2023-08-23

**Authors:** Joshua Baldwin, Joomi Lee, Allan D. Tate, Christian D. Okitondo, Kyle Johnsen, Michael D. Schmidt, Stephen Rathbun, Eric Novotny, Sun Joo (Grace) Ahn

**Affiliations:** 1 Grady College of Journalism and Mass Communication, University of Georgia, Athens, GA 30602-3018, USA; 2 Department of Communication, University of Arkansas, Fayetteville, AR 72701, USA; 3 Department of Epidemiology and Biostatistics, College of Public Health, University of Georgia, Health Sciences Campus, Athens, GA 30602, USA; 4 School of Electrical and Computer Engineering, College of Engineering, University of Georgia, Athens, GA 30602, USA; 5 Department of Kinesiology, Mary Frances Early College of Education, University of Georgia, Athens, GA 30602, USA; 6 Workplace Research and Insights, Haworth, Holland, MI 49423, USA

**Keywords:** social support, sensor-based technologies, children, avatars and agents, health

## Abstract

Sensor-based technologies (SBTs) allow users to track biometric data and feature interactions that foster social support. The social support from SBTs can increase intrinsic motivation to engage in and sustain positive health behaviors. Guided by technological affordances and self-determination theory, this study tested the long-term efficacy of an ecosystem of SBTs to strengthen social support for children’s behavior change, children’s perceived relatedness, and positive physical activity (PA) attitudes. This ecosystem integrated Fitbits tracking each child’s PA, kiosks with virtual agents that synced with Fitbit data, and a messaging system for parents and children. Afterschool programs (*N* = 19) were randomly set with this ecosystem or a Fitbit with a computer for 6 months. Results suggested that parents of girls provided more social support to children via the ecosystem than parents of boys. Children’s perceived support from the virtual agent was positively associated with perceived relatedness and PA attitudes over time.

## Lay Summary

Social support can be a positive force to get children to engage in PA. The current study examined a proprietary digital system that used SBTs (e.g., Fitbits, motion-sensing games) and a computer-generated virtual dog called the virtual fitness buddy (VFB). It was hypothesized that the VFB could complement caregivers’ social support to their children. An experiment randomly put the VFB system or a control alternative at 19 YCMA afterschool programs. Results showed that having the VFB system at a YCMA site prompted caregivers of girls to give a higher amount of social support. It was also observed that the more children thought the virtual pet was supportive, the more they felt supported and had a positive opinion toward PA.

Although individual skills and competence are important in inducing behavior change, earlier work in communication scholarship has emphasized the importance of social support in motivating and sustaining behavior change. Social support generally refers to any communication activity from and with people in one’s support network that is intended to provide emotional, instrumental, or informational help to cope with uncertainty and stress or to promote well-being (Albrecht & Adelman, 1987; Goldsmith & Albrecht, 2011; Meng et al., 2017). The information and resources obtained from social support have been theorized to assist individuals to meet personal goals and experience mastery in the demands of various situations (Bandura, 1976; Eyres & MacElveen-Hoehn, 1983; Ryan & Deci, 2017; Tolsdorf, 1976).

Particularly for children, who are still vulnerable and developing their capacity for self-regulation, social support and communicative feedback from adults in their support network have been shown to play a critical role in the internalization and persistence of health behaviors (Beets et al., 2010; Trost et al., 2003). Within the family, parents can provide tangible and intangible forms of social support to increase children’s physical activity (PA), including logistical support (e.g., transporting the child to afterschool sports), modeling (e.g., exercising as an example), communicating (e.g., giving encouragement), and restricting sedentary activities (e.g., limiting screen time; Davison et al., 2011). However, providing systematic and consistent support to children can be burdensome and untenable for caregivers, especially working parents. Some children may also be living with instability in their homes without reliable access to consistent social support (Brockman et al., 2009).

Sensor-based technologies (SBTs) have been spotlighted for their accuracy and usefulness as tools for monitoring behaviors, particularly in the context of digital health interventions (Morris & Aguilera, 2012). Technical capabilities of SBTs, such as wearables and motion sensors, include automatic collection of interaction data and real-time feedback to users and those close to them. For example, many exergames (i.e., games for promoting exercise-relevant attitudes and behaviors; Peng et al., 2012) integrate SBTs to track player motion through controllers and apply player movements to avatars. These games provide health-related information such as posture and weight (*Wii Fit*) or walking distance per week (*Pokémon Go*). Similarly, wearable devices like the Fitbit can track an assortment of metrics including the user’s number of steps, heart rate, and sleep hours. The immediate feedback from these technologies allows individuals to observe and adjust health-related behaviors.

Less attention has been given to the technological affordances of SBTs as a communication technology that promotes social interactions and amplifies social support. Via sensor-based automation and computer-mediated communication (CMC), SBTs can enhance the frequency, consistency, and reliability of socially supportive communication. For example, SBTs could help parents by providing important metrics about their children’s PA and prompting them to encourage their children to engage in PA and provide necessary resources. Therefore, assessing the extent to which SBTs can foster parental involvement in a child’s daily PA by way of parent–child interaction, communication, and support is warranted.

The current study examined a SBT-based intervention that incorporates interconnected sensors designed to amplify the effectiveness of a child’s social support network. We present evidence that SBTs can promote critical social support behaviors, above and beyond mere data tracking, by enhancing parent–child communication and fostering fundamental shifts in the support structure surrounding the child based on real-time input and feedback.

## Social support and support networks

The effectiveness of social support in behavior change is predicated on the quality of the support network (Meng et al., 2016; Penner et al., 2000). Research in this area suggests that effective support will most likely occur within networks that allow the flow of supportive communication (as opposed to non-supportive communication), have strong relational ties, and encourage positive (i.e., empowering communication) rather than negative social support (i.e., critical communication) (see Goldsmith & Albrecht [2011] for review). Additionally, research applying the network theory of brokerage and closure (Burt, 2005) to support networks suggests that individuals are more likely to receive emotional and logistical support when people close to them are more interconnected with each other (Meng et al., 2016).

Based on this logic, several factors may strengthen a child’s support network. For one, a child’s support network may be more successful if parents and others can provide immediate, just-in-time (rather than delayed) social support. Incentive theory (Killeen & Fetterman, 1988) suggests that a child will be more motivated to engage in a behavior when positive social support is immediately communicated (i.e., when the association between behavior and reward is temporally close). However, children often cannot receive immediate positive support from people within their support network. A large number of U.S. households are dual-income families with both parents in the labor force (U.S. Census Bureau, 2021) and parents may not always be immediately available to provide timely social support due to work and other situational constraints. Households of low socio-economic status (SES) often lack the necessary level of socio-technological infrastructure to provide effective social support for children’s health and education (Katz et al., 2017). Thus, parents may find it difficult to be informed about their child’s PA and to offer immediate feedback.

Moreover, social support is not monolithic and unilaterally positive. Despite best intentions, sometimes feedback to children may not be encouraging or empowering but rather critical and controlling (Goldsmith & Albrecht, 2011). In these cases, social support from the children’s support network may increase stress and uncertainty and decrease their intrinsic motivation to engage in a behavior.

The technological affordances of SBTs may provide ways for parents to stay informed about their child’s daily PA, deliver immediate and timely social support, and deliver consistently positive messages. However, little work has attempted to examine how sensor-mediated communication can foster social support for lasting behavior change within a child’s support network. Some research has demonstrated that the features of CMC (e.g., status updates and direct messaging) can strengthen the quality and effectiveness of a person’s support network (Vitak & Ellison, 2013), but less work has examined whether the features of SBTs can amplify the positive impact of social support in behavior change. The current study aims to explore how the unique characteristics of SBTs may afford increased support network effectiveness, specifically in the context of parent–child relationships.

## Using SBT for behavior change

SBTs provide feedback by tracking and logging an individual’s activities. In particular, earlier research has examined the effectiveness of sensor-mediated communication on children’s health behaviors, including treatments that incorporate wearables (Ridgers et al., 2016), exergames (Peng et al., 2011), and mixed-reality kiosks (Ahn et al., 2015; Johnsen et al., 2014). These findings demonstrate that not only does sensors’ automated feedback provide accurate information about user performance or status, but sensors can also communicate social messages to promote positive interpersonal interactions through computer-mediated channels. SBTs could be particularly viable in leveraging the existing relationships within the user’s support network, including family and peers, by alerting them of real-time user behaviors and encouraging social support. This support, in turn, may increase user enjoyment of using SBTs as well as their intrinsic motivation to engage in health-related behaviors.

Sensor-based wearables, such as Fitbits, are devices that record users’ biometric information, such as heart rate, sleep, and step count, using photoplethysmography sensors, accelerometers, and gyroscopes. Results from research on the effect of wearable interventions on children’s health behaviors have been mixed. One meta-analysis on wearable interventions for children’s PA suggested a positive albeit insignificant effect of PA trackers (Ridgers et al., 2016), whereas other studies have found that providing wearables to children can decrease PA motivations (e.g., Kerner et al., 2019). By comparison, studies have shown that exergames can facilitate light to moderate PA for children (Peng et al., 2011). However, this effect may not be long-lasting as relying on extrinsic rewards from the game is likely insufficient to motivate internalization of regular PA in children (Ahn et al., 2019; Liang & Lau, 2014).

Some work has also examined the effectiveness of sensor-embedded kiosks on health-related behaviors. Unlike exergames, kiosks are typically designed for use in public settings (e.g., hospitals and schools). Earlier studies have looked at kiosks designed to provide specific health-related information (e.g., conversational healthcare agents; Bickmore et al., 2010), and to facilitate specific behaviors like healthy eating (Ahn et al., 2016) or engaging in PA (Ahn et al., 2015; Johnsen et al., 2014). Although these earlier studies examined SBTs’ potential to shape behavioral change, evidence for sustained behavior change in children is lacking. Moreover, less work has explored the potential for SBTs to strengthen a child’s social support network as a mechanism of behavior change.

## Technological affordances of SBT

Social connotations of technological affordances have been widely discussed in the domains of CMC and information communication technologies (Davis & Chouinard, 2016; Evans et al., 2017). The concept of *affordances* was originally proposed by Gibson (1986), referring to action capabilities that are detected as an animal interacts with its environment. Norman (1999) extended the discussion of affordances to technology to consider design implications of technological artifacts to support users’ perceptions of how the object can or should be used.

The discourse on media affordances in communication scholarship focuses on the *relational* properties between users, media features, mediated environment, and users’ situational context. Evans et al. (2017) suggested that media affordances are not technological features nor outcomes, and should have variability on a continuum—that is, affordances exist independent of user perceptions, and users may perceive different levels of affordance from the same technology when constrained by their individual characteristics and situational contexts. Understanding media affordances is important in the context of CMC because affordances can shape how users choose to engage with media and other users through mediated experiences.

We posit that the process through which SBTs afford social support for children to ultimately motivate PA is also relational, shaped by the media features of different SBTs and users’ agency in choosing how to leverage these features. Thus, examining the relational properties between a child’s support network and the media features provided by SBTs can provide insights to how technology can amplify a support network’s effectiveness. Applying Goldsmith and Albrecht’s (2011) description of a successful support network, SBTs can aid in the transmission of supportive information (over non-supportive information) through its ability to collect a child’s biometric information that prompts those in the support network to provide supportive feedback. SBTs can also strengthen the relational ties between the child and those in the network by facilitating communication between the child and others. Finally, SBTs can cultivate positive social support through virtual agents programmed to mimic social support that can be commonly found in support networks.

Grounded in the idea that affordances vary user experiences on a continuum, Davis and Chouinard (2016) outlined several mechanisms of how technological affordances operate: request, demand, allow, encourage, discourage, and refuse. Applying these dimensions to SBTs delineated how each technological feature could strengthen a support network’s effectiveness. Notably, the biometric sensors *allow* real-time tracking of children’s PA data and sharing feedback with their parents, thereby *encouraging* parents to pay attention to realtime data and respond with social support. Similarly, virtual agents within sensor-based games may *encourage* a child to play and interact which *allows* children to connect with additional support sources in a positive light. Finally, sensor-based communication channels may *request* immediate feedback from parents and *discourage* delayed support. Together, these affordances define how complementing technological capabilities found within an interconnected system of SBTs could strengthen a child’s support network.

Rather than focusing on the features of individual SBTs, as has been the case in earlier research, we suggest an affordance-based multi-channel approach, wherein multiple interconnected SBTs (Fitbits, a kiosk, and parents’ mobile/smartphone) create an *ecosystem* that affords children’s social interactions with their parents (human–human) and virtual agents (human–machine), as well as connections among SBTs. The primary goal of the SBT intervention was to afford parents the ability to provide social support to their children remotely via the feedback system and for children to receive reliable social support. By tracking children’s PA through Fitbits and having parents communicate to the kiosk through their smartphone, parents could support a child’s PA goals by providing encouragement, positive feedback, and advice even if the parent could not be co-located with the child. Therefore, the SBT intervention was anticipated to increase parental social support:

**H1**: Parental support for a child’s PA will increase at a higher rate over time for families in the SBT intervention condition compared with families in the control condition.

## Satisfying psychological needs with SBT

To date, most SBT interventions have relied on features that provide extrinsic rewards by focusing on accurate data tracking and quantified feedback (Godino et al., 2020; Kerner et al., 2019). However, because promoting behavior through extrinsic rewards can undermine intrinsic motivation for the behavior (Deci et al., 2001; Ryan & Deci, 2017), design decisions must consider fostering children’s intrinsic motivation (i.e., motivation driven by internal goals for pleasure) to ensure sustained behavioral change (Peng et al., 2012). Considering earlier findings that social support is inherently linked with intrinsic need satisfaction (Ryan & Solky, 1996), scholars can leverage SBTs’ utility to amplify social support to foster motivations above and beyond quantified feedback.

Self-determination theory (Ryan & Deci, 2017) stipulates that humans have universal psychological needs that, when fulfilled, can drive behavior change that is enjoyable and sustainable. The three fundamental psychological needs that drive intrinsic motivations are competence (the need for achievement and ability to obtain one’s goals), autonomy (the need to freely make choices), and relatedness (the need to feel belonged, valued, and close to others). Although these psychological needs are universal and innate, they can be strengthened or weakened by one’s social and physical environments.

Because children, particularly prior to the onset of adolescence, are still dependent on caregivers for physical and social resources (e.g., Bowlby, 1969) and learning how to form interpersonal relationships with peers (e.g., Howes, 1987), fulfilling the psychological need for relatedness is especially important for sustained behavior change. Earlier studies have demonstrated that fulfilling psychological needs has a small-to-moderate positive effect on PA (Owen et al., 2014), and that social support from others contributes to satisfying the need for relatedness, which subsequently strengthens intentions to engage in PA (George et al., 2013).

Marrying the affordances-based perspective with self-determination theory would suggest that when people perceive behavioral possibilities to fulfill their psychological needs from technology features (i.e., affordances), they will seek out and engage in the behavior. In the context of PA, the features of SBTs can increase the perceived ability to satisfy needs for autonomy (e.g., the degree of customization) and competence (e.g., goal difficulty; Peng et al., 2012). Some preliminary work has suggested that SBTs can bolster relatedness needs to engage in PA through social support (Tsai et al., 2021).

Using SBTs to increase the effectiveness of a child’s support network by enhancing the speed and reliability of social support may be especially potent in associating children’s PA behaviors with the fulfillment of relatedness needs (Ryan & Solky, 1996). As children receive social support from their parents through SBTs (e.g., encouragement through text messages, providing logistical support when using the Fitbit), they should feel increased satisfaction of their perceived relatedness needs, with children feeling supported by their parents. The SBT-enhanced social support from parents to children regarding PA is also anticipated to shed a favorable light on PA (Tsai et al., 2021). Therefore, we hypothesized:

**H2**: Children in the SBT intervention condition will report greater (A) satisfaction of relatedness and (B) positive attitude toward PA compared with children in the control condition.

**H3**: Parental social support will be positively related to the child’s (A) relatedness satisfaction and (B) the child’s positive attitude to PA.

In addition to the SBT intervention’s ability to mediate parent-child communication, virtual agents can also be designed to provide support (Norouzi et al., 2022) during the moments when parents cannot be available. The computers are social actors (CASA) framework suggests that individuals often respond to virtual agents similarly to how they would respond to others in real life (Gambino et al., 2020; Reeves & Nass, 1996). This framework argues that users will heuristically employ social scripts when engaging with virtual entities containing social and autonomous cues (Nass & Moon, 2000). However, these heuristics may decrease as one becomes familiar with the virtual agent (Gambino et al., 2020), and other mechanisms, such as social support, may become more relevant for sustaining behavioral change for the long term.

Child development research indicates that children judge the quality of friendship based on distinct dimensions that include play, help, security, closeness, and low conflict (Bukowski et al., 1994). A virtual agent with these interpersonal qualities may continue to be viewed as supportive over time even as heuristic social scripts become less relevant for sustaining behavioral change. The virtual agent in the current intervention was designed to take on the appearance of a medium-sized dog to minimize perceptions of conflict or criticism that may arise from receiving feedback from peers or adults (Norouzi et al., 2022). The virtual pet helped children set self-determined goals, provided consistent and immediate encouragement based on SBT data (Fitbit), played with children through SBTs embedded in the kiosk (motion-tracking and touch screen), and provided reinforcement (Ball et al., 2021). Therefore, we anticipated:

**H4**: Within the SBT intervention condition, children will perceive increased social support from the virtual agent for their PA over time.

Moreover, as the virtual agent provides social support through feedback and play, children are anticipated to build rapport and feel responsible for their pets’ care, similar to a real-life dog (Donath, 2004). The interactions, in turn, should encourage children to form attachments and fulfill relatedness needs through virtual agents. Consequently, this attachment may allow children to associate intrinsic rewards through PA:

**H5**: Perceived social support from the virtual agent will be positively related to the child’s (A) relatedness satisfaction and (B) the child’s positive attitude to PA.

Lastly, previous research has observed that boys are more likely than girls to receive social support for their PA from parents (Beets et al., 2006), and adolescent girls are less likely to engage in PA than boys (Merlo et al., 2020). This underscores the importance of developing a PA intervention that appeals to young girls and their parents. However, it is unclear whether this sex disparity in social support for PA can be addressed through SBT-based interventions. Earlier research has observed that relatedness motivation is associated with PA attitudes for girls (e.g., Shen et al., 2012). Thus, an SBT intervention with features to satisfy relatedness needs through fostering social support for PA may be particularly appealing for girls. Nevertheless, little to no empirical work has examined sex-based differences of SBTs’ impact on PA attitudes and parental support. We examined the following research question:

**RQ1:** Will a child’s sex moderate the effect of the SBT intervention on parental social support for a child’s PA, perceived relatedness, and positive attitude toward PA?

## Methods

**Figure 1.**
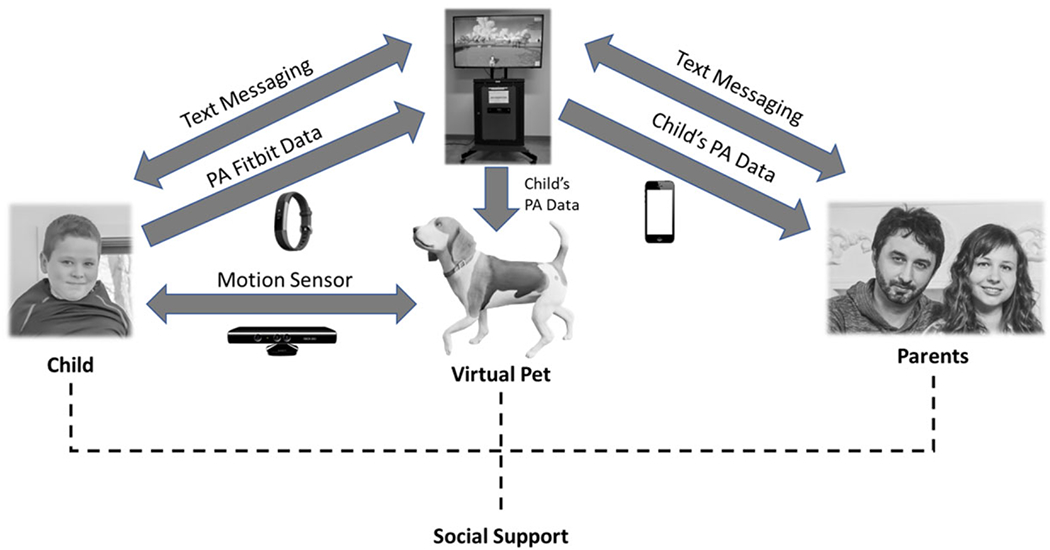
The VFB ecosystem. *Notes*. The VFB ecosystem contained several SBTs designed to afford social support from parents and the virtual pet to the child. The child wore a Fitbit, with the PA data uploaded to a kiosk set at the afterschool program. The PA data influenced how a child played and interacted with their virtual pet using the Kinect motion sensor embedded in the kiosk. The child’s interaction with the kiosk triggered real-time text messages from the virtual pet to the parents to inform them of the child’s PA progress, and parents could send text messages to their child while they used the kiosk.

The present report is a part of a larger community intervention using SBTs and virtual agents to increase children’s PA (Hahn et al., 2020a, 2020b). Specifically, this study tested the efficacy of a system of interconnected SBTs labeled the virtual fitness buddy (VFB) ecosystem. The virtual agent in the ecosystem was presented through a mixed-reality kiosk. The kiosk was a rolling stand that consisted of a Dell Inspiron 5680 computer connected to a 55-inch display, a Microsoft Kinect motion sensor, and an LCD touchscreen (Figure 1). The kiosk contained a proprietary application where children were given a customizable virtual dog that they could name and personalize (e.g., breed, collar, and tag). Children wore a Fitbit during all waking hours throughout the study to track PA. The kiosk downloaded the PA data and the child’s virtual pet synced with the tracked PA data to assist the child in setting a new PA goal and to provide feedback, social support, relationship building through play, and vicarious reinforcement (Ball et al., 2021). As such, the VFB ecosystem is a mediated system of SBTs that provide social cues to a support network through biometric monitoring, communication channels, and game mechanics.

By engaging in PA recorded by the Fitbit, participants could earn credits that they used to dress their dog, buy toys, and unlock new motion games. In line with self-determination theory, participants could set their own PA goals, without an externally designated goal. As participants engaged in more PA, their virtual dog became fitter, faster, and had more stamina to play games. The software featured a messaging system where children could send messages to their parents’ phones and receive supportive responses from parents while interacting with the virtual pet.

### Participants

Two cohorts of families participated in the study for 9 months (cohort 1) and 6 months (cohort 2). Data from the 9-month time point of cohort 1 are not used in the current study, stemming from the inability to collect data at the same 9-month time point with cohort 2 due to the COVID-19 pandemic. Participating children between the ages of 6–11 (grades 1–5; *N*_cohort 1_ = 253; *N*_cohort 2_ = 162) and one primary caregiver for each child were recruited via announcements posted at YMCA-based afterschool programs in the Southeast (12 sites for cohort 1 and 7 sites for cohort 2).

For this article, only self-report measures from children ages 8–11 (*N*_cohort 1_ = 169; *N*_cohort 2_ = 110; *M*_age_ = 8.87, *SD*_age_ = 0.85; 44.09% female; 40.14% White; 25.81% African American; 11.1% South Asian; 4.30% Hispanic/Latino; 3.23% East Asian) and all parents (including those with younger children; *M*_age_ = 40.71, *SD*_age_ = 6.70; 77.83% female; 40.96% White; 30.36% African American; 9.88% South Asian; 6.51% Hispanic/Latino; 4.34% East Asian) were examined. In total, 46.51% of families had an income of $100,000 or more with 62.17% of parents having either a bachelor’s or master’s degree.

Intraclass correlation coefficients (ICCs) were computed to examine how much variability was within children nested within schools across 6 months. At the child within-school level, examination of ICCs revealed that correlated measures across time were primarily at the child level, ranging from 0.32≤ICC≤0.71 (Table 1). Correlated errors across sites were low (ICC < 0.03).

### Procedures

A group-randomized longitudinal design was used in which afterschool programs were randomly assigned to either the VFB ecosystem treatment or a control condition. At the treatment sites, the VFB kiosk was set up in the building of the afterschool program and children were given a Fitbit worn on the wrist that could sync PA data with the kiosk. At the control sites, a standard computer without the virtual pet or the SBT-embedded kiosk was set up. Children in the control condition were still able to sync their PA activity data from their Fitbit with the computer and receive accurate SBT readings, as well as set their own PA goals. However, they were unable to interact with their caregiver or the virtual pet. Randomization was completed by matching pairs of afterschool programs based on similarity in the school’s free and reduced lunch percentage (i.e., a measure of SES) and randomly placing each school in the pair in either the treatment (*n*=10) or control condition (*n*=9).

At baseline, researchers set up kiosks, distributed Fitbits, and administered the first wave of surveys. Site staff were instructed on how to operate the kiosk or standard computer at each afterschool day throughout the intervention. Children were introduced to the kiosk and the virtual pet (treatment group) or a standard computer (control group) and received Fitbits to track PA progress. Parents were asked to attend orientation sessions to learn how to communicate with and support their children using text messages (treatment group) or standard information about the intervention (control group). Following the baseline, children and parents completed three more sessions of surveys assessing their attitudes toward PA, once every 3 months to track changes over time. Different surveys were administered to children and their parents. During the study, children and parents were free to use the kiosk and Fitbit (treatment group) or the standard computer (control group) as much as they liked. All procedures were approved by the University of Georgia Institutional Review Board and preregistered on ClinicalTrials.gov (NCT03524183).

### Measures

#### Parents’ support of child’s PA

To measure parental support toward their child’s PA, parents responded to 16 items from the Activity Support Scale for multiple groups (Davison et al., 2011), assessing different forms of parental support for children’s PA on a Likert-type scale (1 = *Never*; 5 = *Always*). The scale included items that measured different dimensions of social support including four items measuring logistical support (e.g., “I take my child to places where they can be physically active.”), four items measuring modeling (e.g., “I include my child when I do something active.”), one item measuring communication (“I communicate with my child about their PA.”), and seven items measuring restricting access to sedentary activities (e.g., “I don’t like my child to hang around indoors when the weather is nice.”).

#### Child’s perceived relatedness

Perceived relatedness satisfaction was measured with four Likert-type items (1 = *Not at all true*; 3 = *Definitely true*) from the Self-Determination Theory Scale for children’s PA (e.g., “I am active because others want me to be active with them.”; Sebire et al., 2013). Only children in the second cohort completed this measure.

#### Perceived virtual pet support of child’s PA

The child’s perception of the virtual pet’s support was measured using four Likert-type items (1=*Never*; 5=*Everyday*) adapted from the Peer Support Scale (Prochaska et al., 2002) and was reworded to apply to a virtual pet (e.g., “My virtual pet tells me that I am good at PA.”). Because children only interacted with the virtual pet within the VFB ecosystem, only those in the treatment condition completed this measure.

#### Child’s attitude toward PA

Finally, children’s attitude toward PA was measured with seven Likert-type items (1=*Not at all true*; 3=*Definitely true*) from the Children’s Attraction to Physical Activity Scale (Brustad, 1993). The scale included two dimensions measuring the extent to which the children liked to exercise with four items (“I like exercising a whole lot.”) and the extent to which the children viewed exercising as important with three items (e.g., “I think it is very important to always be in good shape.”). All measures for children included “*I don’t understand the question*.” which was coded as missing data. Descriptive statistics and Cronbach’s alpha for each measure are reported in Table 2.

### Statistical analysis

We computed descriptive statistics to examine the outcome variables measured over the baseline, 3-months, and 6-months periods. The primary effect of interest was the interaction of treatment group and period (baseline, first follow-up at 3 months, and second follow-up at 6 months). The period effect was operationalized as a continuous random variable to examine a change in treatment group over time of parents’ support of child’s PA, child’s perceived relatedness, and child’s attitude to PA. Multilevel general linear mixed models (MLMs) were fitted with two random intercepts to account for students observed over time and nested within-afterschool which was the unit of randomization. For secondary analyses of sex differences in treatment effects, stratum-specific (girl and boy separately) effects of randomization to the VFB intervention were presented. Pairwise Pearson correlations were computed for all dependent variable pairs at each observation point, and the sex-based interactions of treatment effects were evaluated to examine differences in intervention effects for boys and girls. Statistical significance was set to the 0.05 level and all models were fitted using the {mixed} modeling procedure in Stata 17.0 MP (College Station, TX).

## Results

### Effect of VFB ecosystem on parental support, perceived relatedness, and PA attitudes

The primary aim was to evaluate the effect of randomization to the VFB ecosystem on parental support, child’s perceived relatedness, and child’s PA attitudes over 6 months of followup. There was no evidence that assignment to the VFB treatment had greater effects on all dependent variables across time compared with the control condition after controlling for child sex (all Treatment × Period interactions, *p* > .05).

The secondary aim was to evaluate whether treatment effects were stronger for boys or girls. The sex-based treatment interactions supported that parent provision of social support effects was higher among girls compared with boys. For example, parent provision of social support to girls was *β* = 0.24, 95% confidence interval (CI): [0.06, 0.41] which was stronger than the effect for boys, *β* = 0.04, 95% CI: [−0.09, 0.17] (Table 3). Standardized treatment effect sizes (Cohen’s *d*) were 0.07 for boys and 0.43 for girls indicating moderate-sized treatment effects for girls (*p*=.01). A subdomain analysis indicated that the sex-based interaction on treatment effects was strongest among logistical support (*p*=.02) for girls, *β*=0.41, 95% CI: [0.10, 0.71], compared with boys, *β*=0.13, 95% CI: [−0.02, 0.28]. The interaction effects between sex and treatment on all other primary outcomes were nonsignificant. Hypotheses 1 and 2 were not supported, and the VFB ecosystem treatment did not have observable overall impact on children’s self-reported relatedness needs or their attitudes regarding PA compared with Fitbits and standard computers.

### Perceived pet social support over time

Descriptive analyses of children’s perceived support from their virtual pet during the VFB treatment revealed almost no change between the 3-month (*M*=3.00, *SD*=1.25, *n* = 104, 95% CI: [2.77, 3.24]) and 6-month measurement periods (*M*=3.08, *SD*=1.31, *n* = 84, 95% CI: [2.80, 3.36]). Children in the treatment condition *sometimes* felt that their virtual pet was supportive and this view remained stable throughout the study. Hypothesis 4 was not supported.

### Relationship between social support, perceived relatedness, and PA attitudes

Hypotheses 3 and 5 were examined using Pearson product correlations to evaluate bivariate associations between parental support and the child’s perceived pet support, relatedness needs satisfaction, and the child’s attitude to PA (liking and perceived importance) within each condition and time period (Table 4). In the control condition, there appeared to be evidence that child attitudes toward PA as well as relatedness (psychological needs satisfaction) were correlated with parental support (*p* < .05). The strongest associations were observed at the 3- and 6-month observation periods within the control condition. There was no strong evidence of these bivariate relationships among the treatment arm.

Finally, we inspected the relationship between a child’s perception that the virtual pet provided social support, and their relatedness need satisfaction and attitude toward PA. Because only children in the VFB treatment condition interacted with a virtual pet, only correlations within the treatment condition were available. Results indicated that perceived pet support was significantly and positively related to a child’s relatedness satisfaction (*r* = 0.37, *n*=41, *p* = .02) and liking PA (*r* = 0.31, *n*=103, *p* < .001) at the 3-month period. Additionally, at the 6-month time period, the relationship between perceived pet support and PA importance was significant (*r* = 0.28, *n*=84, *p* = .01) while the relationship between perceived pet support and PA liking fell just outside of significance (*r* = 0.21, *n*=84, *p* = .06).

## Discussion

SBTs allow users to accurately and immediately monitor and record biometric data (Morris & Aguilera, 2012). However beyond accurate data tracking, emerging scholarship suggests that SBTs may serve as a potent *mediator* of social interactions that can amplify the effectiveness of a support network. We demonstrated in the current study that SBTs can connect users and help them move toward a common, shared goal, even when they are not colocated in the same space. SBT-mediated interactions also fostered relationships between children and virtual agents that served as complementary nodes in the support network. Despite existing views of SBTs as devices that merely track and report data, SBTs may play a central role in communicating and mediating social support that shapes sustained behavioral outcomes.

### Facilitating parental social support for girls versus boys

Our results demonstrated that the VFB ecosystem can garner social support from parents, but parents of boys and girls responded differently. The VFB ecosystem was especially effective at fostering parental support for girls over time. Previous research has suggested that while parental support is similarly effective at fostering PA for girls as for boys (Laird et al., 2016), girls are less likely to receive parental support (e.g., Beets et al., 2006). This could account for the sex differences observed in PA in adolescence, with boys engaging in markedly more PA than girls (Merlo et al., 2020).

From a technological affordance perspective (Evans et al., 2017), the increased capacity of social support emerged from the relationship between the characteristics of families and the media features found within the SBT ecosystem. Here, we observed that caregivers of girls were more willing to provide increased support when exposed to the SBT intervention, and that this increase in support was sustained for at least 6 months. The SBTs in the ecosystem may have steadily signaled parents of girls about their child’s PA performance, or the lack thereof. If so, parents of girls may have felt a greater responsibility to provide logistical support so that their child could use the VFB ecosystem regularly. In the ecosystem, the SBTs *requested* parents to stay updated regarding their children’s PA progress and *encouraged* parents to find activities that required PA while *discouraging* parents from having their children engage in sedentary behavior (Davis & Chouinard, 2016). The relational ties between child and caregivers within a support network may have been strengthened by these interactions within the SBT ecosystem.

This finding is encouraging as support from parents has been shown to increase a child’s PA and has been a barrier for girls to engage in PA, particularly as they grow into older childhood (Beets et al., 2010; Trost et al., 2003). However, given parents’ situational constraints (e.g., workload and lack of technology infrastructure) that prevent them from providing immediate social support to their child (Katz et al., 2017), the current findings indicate that SBTs can mediate timely parental support (Killeen & Fetterman, 1988), and seems particularly effective for parents of girls, addressing a well-known critical barrier to PA in children.

Based on our observations, we proffer some practical recommendations when administering a digital PA intervention for girls. First, the intervention should employ the benefits of SBTs in mediating supportive communication between children and those in their support network. Our SBT ecosystem leveraged several different interconnected technologies (e.g., wearables, kiosks, and smartphones) that could mediate support between the child and other nodes in the support network. Although the current study explored how SBTs can strengthen the relational ties between the child, caregiver, and virtual agents within a support network, other ties could also be strengthened through SBT-mediated channels such as peers and teachers. For instance, children could share or trade virtual rewards they earned from PA tracked by wearables with their peers. We would expect adding digital channels to communicate with other relational ties to be especially appealing for girls seeking relatedness fulfillment (Shen et al., 2012).

### Facilitating social support through virtual pets

Findings in the CASA paradigm (Gambino et al., 2020; Reeves & Nass, 1996) have indicated that people interact with virtual agents as if they were social entities and may respond to their actions similarly. However, these heuristic interactions with a virtual agent are not long-lasting (Gambino et al., 2020); therefore, additional considerations are needed when designing a virtual agent for long-term influence. Current findings indicate that one way this could be achieved is through supportive interactions between users and virtual agents. An individual’s ongoing interactions with virtual agents that convey plausible feedback can increase rapport that is characterized by mutual trust and coordination, which is essential for psychological need satisfaction and motivation to achieve behavioral goals (Baker et al., 2020; Olafsson et al., 2020). The virtual agent in the present study was designed to form personalized social bonds with children through real-time interactions as well as social partnership to achieve PA goals together via positive reinforcement (Ahn et al., 2015), contingent upon the child’s PA performance tracked through SBTs.

Children perceived moderate but steady levels of social support from the virtual agent throughout the 6 months of the study, demonstrating that a virtual pet served as an agent of social support. Earlier research demonstrated that a virtual agent can socially support and encourage adults to engage in health-related behaviors by providing useful information (Bickmore et al., 2010). However, little empirical work has tested the viability of prolonged relationships between users and virtual agents. Our investigation suggests that similar to how people develop long-term relationships with their real pets, children may have perceived reciprocal support from their virtual pets based on the range of interactions with the features afforded by the virtual pet. Despite the limited amount of preprogrammed responses that the pet could provide, children reported perceiving steady social support from the virtual pet across 6 months, implying that consistency and reliability of social support may be more important than the novelty of supportive content. The findings indicate that virtual agents can fulfill intrinsic needs of children by building rapport through on-demand play and reliably positive feedback.

### Relationship between social support, relatedness needs, and attitudes

In line with self-determination theory (Ryan & Deci, 2017) and social cognitive theory (Bandura, 1976), we predicted that parental support would be intrinsically rewarding and pleasurable to the child and should help the child form positive attitudes toward behaviors associated with that reward (i.e., PA). Contrary to expectations, relationships between parental support and these outcomes were only positive and statistically significant within the control condition. By comparison, in the VFB ecosystem condition, children’s perceived virtual pet support was positively associated with perceived relatedness and PA attitudes.

There may be several explanations to these results. First, children who had a virtual pet may have thought that its consistent support satisfied their relatedness needs more than the support received from parents. Alternatively, the lack of expected findings might be caused by ceiling effects or subject attrition within the VFB treatment condition, leading to smaller sample sizes compared with the control condition. It is also likely that the increase in treatment effect in parental support for girls and the decrease in parental support for boys washed out measurable treatment effects. Future research is needed to determine these alternative explanations.

### Limitations

There are limitations that qualify the generalizability of current findings. First, our sample consisted of families who were relatively higher in SES. Previous research has suggested that children from higher SES are more likely to engage in PA than children from lower SES (Drenowatz et al., 2010). Thus, it may be possible that the VFB intervention was implemented with children with existing high PA and low sedentary behaviors, resulting in a ceiling effect wherein the impact of the VFB ecosystem can seem negligible. A sample of families and children from lower SES may demonstrate stronger treatment effects. Second, although the VFB ecosystem was designed to be easily managed and run by afterschool staff, the degree to which each location implemented the kiosks between sites varied (e.g., some had the kiosk out every day while others did not). This implementation inconsistency may have weakened the observed treatment effect and attenuated our ability to observe differences between conditions. Third, it might be the case that the insignificant treatment by period interaction effect on parental support was due to the inability of the VFB ecosystem to increase parental support for boys, washing out the positive impact of the treatment on parental support for girls. Fourth, as with most longitudinal designs, we had a substantial rate of participant attrition after the 3- and 6-month periods. Loss to follow-up may have introduced selection bias in our follow-up that should be considered when interpreting our findings.

## Conclusion

SBTs not only provide immediate and accurate biometric information for users, but also facilitate social cues that afford social support created through positive interpersonal interactions. The present investigation developed an SBT-integrated ecosystem that supports such interactions by leveraging technological affordances of SBTs to foster social support for children and fulfill relatedness needs via a virtual agent. We found evidence that social support can be espoused from interconnected SBTs and that the sex of the child moderated this outcome. Given that social support can be critical in sustaining PA over the long term, future research should determine the extent to which such an intervention can be generalized, how different technological affordances of different SBTs contribute to amplifying social support (e.g., virtual agent, communication channel, and motion-detection), and how social support needs differ among boys, girls, and their parents. SBT-based interventions, such as the VFB ecosystem, have the potential to complement a child’s existing support network at a fraction of the cost and labor involved. Notably, our intervention was administered in 19 afterschool sites to hundreds of children for at least 6 months, demonstrating the VFB ecosystem’s feasibility, scalability, and sustainability despite implementation challenges outside of the laboratory. This study provides an initial step in better understanding the ability of a holistic SBT ecosystem to create a support environment conducive to lasting behavior change.

## Supplementary Material

Supplementary materials

## Figures and Tables

**Table 1.** Child within-school intraclass correlations of outcome measures

	Parental social support	Virtual pet social support	Relatedness	PA liking	PA importance
Intraclass correlation	0.71	0.54	0.46	0.51	0.31

**Table 2.** Descriptive statistics of outcome measures

	Baseline				3-months				6-months			
	*N*	*M*	*SD*	ɑ	*N*	*M*	*SD*	ɑ	*N*	*M*	*SD*	ɑ
Parents’ social support	389	3.99	0.60	0.84	295	3.98	0.53	0.82	251	4.01	0.55	0.83
• Logistical support	392	4.02	0.87	0.82	295	3.96	0.83	0.83	251	4.05	0.84	0.82
• Modeling	392	3.79	0.86	0.82	295	3.73	0.74	0.77	251	3.73	0.78	0.82
• Communication	390	3.86	0.99	–	295	3.86	0.87	–	251	3.88	0.91	–
• Restricting SA	389	4.07	0.61	0.64	295	4.13	0.56	0.65	251	4.13	0.58	0.66
Virtual pet’s social support	–	–	–	–	104	3.00	1.25	0.89	84	3.08	1.31	0.91
Perceived relatedness	105	2.62	0.37	0.63	106	2.56	0.44	0.77	89	2.60	0.44	0.82
PA liking	269	2.25	0.41	0.52	247	2.21	0.42	0.55	210	2.27	0.39	0.45
PA importance	266	2.75	0.35	0.52	248	2.71	0.40	0.58	210	2.73	0.37	0.53

*Note*. SA = sedentary activities.

**Table 3.** Mixed effect model examining the interaction of child sex-treatment effects by girl/boy strata

Outcomes	*β*-girls treatment effect (95% CI)	*β*-boys treatment effect (95% CI)	Interaction *p* value
Parents’ social support	0.24 (0.06, 0.41)**	0.04 (−0.09, 0.17)	**.01**
• Logistical support	0.41 (0.10, 0.71)**	0.13 (−0.02, 0.28)	**.02**
• Modeling	0.25 (−0.01, 0.51)	0.03 (−0.14, 0.20)	.09
• Communication	0.25 (−0.02, 0.53)	0.04 (−0.16, 0.24)	.11
• Restricting SA	0.13 (−0.02, 0.28)	−0.01 (−0.18, 0.16)	.11
Perceived relatedness	0.08 (−0.05, 0.21)	0.06 (−0.10, 0.21)	.86
PA liking	0.08 (−0.05, 0.22)	−0.02 (−0.13, 0.10)	.29
PA importance	−0.02 (−0.11, 0.07)	0.01 (−0.05, 0.08)	.63

*Note*. SA = sedentary activities.

* *p*<.05;

** *p*<.01;

*** *p*<.001.

**Table 4.** Correlations or parental social support of child’s PA and psychological need satisfaction and PA attitude within condition and time period

Parental social support						
Within treatment						
	Baseline		3 months		6 months	
	*N*	*r*	*N*	*r*	*N*	*r*
Perceived relatedness	42	−0.12	30	0.22	25	0.27
PA liking	115	0.02	83	0.07	68	−0.02
PA importance	112	−0.03	84	0.18	68	−0.22
Within control						
	Baseline		3 months		6 months	
	*N*	*r*	*N*	*r*	*N*	*r*

Perceived relatedness	62	**0.24** *	42	**0.37** **	38	**0.41** **
PA liking	138	0.32****	104	**0.25** **	90	**0.32** ***
PA importance	138	0.06	104	**0.24** **	90	**0.25** **

*Notes*. Measures of autonomy, competence, and relatedness were only measured from children in the second cohort. The full six correlation tables within each condition and time period can be found in the Supplementary Materials.

† *p* < .06;

* *p* < .05;

** *p* < .01;

*** *p* < .001.

## Data Availability

The data used for reported results in this article are available at the Open Science Foundation repository: https://osf.io/zqmcb/?view_only=956f967c1b544a86b37c8cad629c8655.
