# Supplementary material for: Mediating social support through sensor-based technologies for children’s health behavior change
Source: J Comput Mediat Commun. Author manuscript; Available in PMC 2025 Aug 5. (PMC12323752; doi:10.1093/jcmc/zmad011)
Supplement: Supplementary materials [file NIHMS2033179-supplement-Supplementary_materials.pdf]

## Supplemental Materials

## Appendix A: Correlations Between Outcome Variable within Condition and Time Period

### Table 1

| Control-Baseline | | 1 | 2 | 3 | 4 | 5 | 6 | 7 | 8 | 9 | 10 |
|---|---|---|---|---|---|---|---|---|---|---|---|
| 1. Parent Support | r | 1 | | | | | | | | | |
| | N | 213 | | | | | | | | | |
| 2. Logistic Support | r | .800*** | 1 | | | | | | | | |
| | N | 213 | 214 | | | | | | | | |
| 3. Modeling | r | .799*** | .573*** | 1 | | | | | | | |
| | N | 213 | 214 | 214 | | | | | | | |
| 4. Communication | r | .577*** | .411*** | .490*** | 1 | | | | | | |
| | N | 213 | 213 | 213 | 123 | | | | | | |
| 5. Restricting SA | r | .850*** | .466*** | .492*** | .509*** | 1 | | | | | |
| | N | 213 | 213 | 213 | 212 | 123 | | | | | |
| 6. Autonomy | r | -.027 | -.071 | .048 | .146 | -.028 | 1 | | | | |
| | N | 63 | 63 | 63 | 63 | 63 | 64 | | | | |
| 7. Competence | r | .224 | .289* | .245 | .207 | .080 | .365** | 1 | | | |
| | N | 63 | 63 | 63 | 63 | 63 | 64 | 64 | | | |
| 8. Relatedness | r | .244 | .240 | .201 | .250* | .172 | .364** | .441*** | 1 | | |
| | N | 62 | 62 | 62 | 62 | 62 | 63 | 63 | 63 | | |
| 9. PA Liking | r | .320** | .323** | .188* | .264** | .251** | .369** | .610*** | .406*** | 1 | |
| | N | 138 | 139 | 139 | 138 | 138 | 64 | 64 | 63 | 148 | |
| 10. PA Importance | r | .058 | .077 | .091 | .136 | .006 | .393** | .336** | .100 | .146 | 1 |
| | N | 138 | 139 | 139 | 138 | 138 | 64 | 64 | 63 | 148 | 148 |

### Table 2

| Control-3-Months | | 1 | 2 | 3 | 4 | 5 | 6 | 7 | 8 | 9 | 10 |
|---|---|---|---|---|---|---|---|---|---|---|---|
| 1. Parent Support | r | 1 | | | | | | | | | |
| | N | 163 | | | | | | | | | |
| 2. Logistic Support | r | .797*** | 1 | | | | | | | | |
| | N | 163 | 163 | | | | | | | | |
| 3. Modeling | r | .698*** | .483*** | 1 | | | | | | | |
| | N | 163 | 163 | 163 | | | | | | | |
| 4. Communication | r | .590*** | .395*** | .446*** | 1 | | | | | | |
| | N | 163 | 163 | 163 | 163 | | | | | | |
| 5. Restricting SA | r | .828*** | .434*** | .328*** | .523*** | 1 | | | | | |
| | N | 163 | 163 | 163 | 163 | 163 | | | | | |
| 6. Autonomy | r | -.093 | -.009 | -.058 | .121 | -.121 | 1 | | | | |
| | N | 42 | 42 | 42 | 42 | 42 | 65 | | | | |

| | | | | | | | | | | | |
|---|---|---|---|---|---|---|---|---|---|---|---|
| 7. Competence | r | .426** | .604*** | .221 | .320* | .256 | .303* | 1 | | | |
| | N | 42 | 42 | 42 | 42 | 42 | 65 | 65 | | | |
| 8. Relatedness | r | .373* | .403** | .372* | .408** | .214 | .335** | .522*** | 1 | | |
| | N | 42 | 42 | 42 | 42 | 42 | 64 | 64 | 64 | | |
| 9. PA Liking | r | .250* | .299** | .078 | .074 | .180 | .225 | .570*** | .427*** | 1 | |
| | N | 104 | 104 | 104 | 104 | 104 | 65 | 65 | 64 | 139 | |
| 10. PA Importance | r | .238* | .257** | .059 | .217* | .201* | .402*** | .345* | .097 | .336*** | 1 |
| | N | 104 | 104 | 104 | 104 | 104 | 65 | 65 | 64 | 139 | 139 |

## Table 3

| Control-6-Months | | 1 | 2 | 3 | 4 | 5 | 6 | 7 | 8 | 9 | 10 |
|---|---|---|---|---|---|---|---|---|---|---|---|
| 1. Parent Support | r | 1 | | | | | | | | | |
| | N | 142 | | | | | | | | | |
| 2. Logistic Support | r | .799*** | 1 | | | | | | | | |
| | N | 142 | 142 | | | | | | | | |
| 3. Modeling | r | .704*** | .472*** | 1 | | | | | | | |
| | N | 142 | 142 | 142 | | | | | | | |
| 4. Communication | r | .575*** | .397*** | .452*** | 1 | | | | | | |
| | N | 142 | 142 | 142 | 142 | | | | | | |
| 5. Restricting SA | r | .839*** | .464*** | .348*** | .498*** | 1 | | | | | |
| | N | 142 | 142 | 142 | 142 | 142 | | | | | |
| 6. Autonomy | r | .453** | .469** | .198 | .388* | .385* | 1 | | | | |
| | N | 39 | 39 | 39 | 39 | 39 | 56 | | | | |
| 7. Competence | r | .458** | .470** | .427** | .282 | .269 | .299* | 1 | | | |
| | N | 38 | 38 | 38 | 38 | 38 | 55 | 55 | | | |
| 8. Relatedness | r | .411* | .257 | .409* | .446** | .339* | .438*** | .453*** | 1 | | |
| | N | 38 | 38 | 38 | 38 | 38 | 55 | 55 | 55 | | |
| 9. PA Liking | r | .319** | .336** | .146 | .123 | .246* | .211 | .627*** | .340* | 1 | |
| | N | 90 | 90 | 90 | 90 | 90 | 56 | 55 | 55 | 121 | |
| 10. PA Importance | r | .245* | .357** | .145 | .007 | .093 | .248 | .499*** | .192 | .451*** | 1 |
| | N | 90 | 90 | 90 | 90 | 90 | 56 | 55 | 55 | 121 | 121 |

## Table 4

| Treatment-Baseline | | 1 | 2 | 3 | 4 | 5 | 6 | 7 | 8 | 9 | 10 |
|---|---|---|---|---|---|---|---|---|---|---|---|
| 1. Parent Support | r | 1 | | | | | | | | | |
| | N | 176 | | | | | | | | | |
| 2. Logistic Support | r | .776*** | 1 | | | | | | | | |
| | N | 176 | 178 | | | | | | | | |
| 3. Modeling | r | .832*** | .630*** | 1 | | | | | | | |
| | N | 176 | 178 | 178 | | | | | | | |

| | | | | | | | | | | | |
|---|---|---|---|---|---|---|---|---|---|---|---|
| 4. Communication | r | .616*** | .438*** | .516*** | 1 | | | | | | |
| | N | 176 | 177 | 177 | 177 | | | | | | |
| 5. Restricting SA | r | .810*** | .374*** | .450*** | .520*** | 1 | | | | | |
| | N | 176 | 176 | 176 | 176 | 176 | | | | | |
| 6. Autonomy | r | .016 | -.001 | -.057 | -.080 | .072 | 1 | | | | |
| | N | 42 | 42 | 42 | 42 | 42 | 42 | | | | |
| 7. Competence | r | .223 | .119 | .160 | .138 | .273 | .478** | 1 | | | |
| | N | 42 | 42 | 42 | 42 | 42 | 42 | 42 | | | |
| 8. Relatedness | r | -.123 | -.257 | -.079 | -.102 | -.035 | .308* | .241 | 1 | | |
| | N | 42 | 42 | 42 | 42 | 42 | 42 | 42 | 42 | | |
| 9. PA Liking | r | .019 | .3131 | -.026 | -.142 | -.038 | .314* | .471** | .031 | 1 | |
| | N | 115 | 116 | 116 | 116 | 115 | 42 | 42 | 42 | 121 | |
| 10. PA Importance | r | -.027 | -.006 | -.033 | -.074 | -.012 | .373* | .206 | .021 | .395*** | 1 |
| | N | 112 | 113 | 113 | 113 | 112 | 42 | 42 | 42 | 118 | 118 |

## Table 5

| Treatment-3-Months | | 1 | 2 | 3 | 4 | 5 | 6 | 7 | 8 | 9 | 10 | 11 |
|---|---|---|---|---|---|---|---|---|---|---|---|---|
| 1. Parent Support | r | 1 | | | | | | | | | | |
| | N | 132 | | | | | | | | | | |
| 2. Logistic Support | r | .758*** | 1 | | | | | | | | | |
| | N | 132 | 132 | | | | | | | | | |
| 3. Modeling | r | .759*** | .490*** | 1 | | | | | | | | |
| | N | 132 | 132 | 132 | | | | | | | | |
| 4. Communication | r | .524*** | .370*** | .533*** | 1 | | | | | | | |
| | N | 132 | 132 | 132 | 132 | | | | | | | |
| 5. Restricting SA | r | .803*** | .369*** | .359*** | .341*** | 1 | | | | | | |
| | N | 132 | 132 | 132 | 132 | 132 | | | | | | |
| 6. Pet Support | r | .017 | -.030 | .037 | .215 | .028 | 1 | | | | | |
| | N | 80 | 80 | 80 | 80 | 80 | 104 | | | | | |
| 7. Autonomy | r | -.287 | -.174 | -.282 | -.153 | -.228 | .108 | 1 | | | | |
| | N | 30 | 30 | 30 | 30 | 30 | 41 | 42 | | | | |
| 8. Competence | r | .154 | .221 | -.125 | -.024 | .215 | .120 | .084 | 1 | | | |
| | N | 30 | 30 | 30 | 30 | 30 | 41 | 42 | 42 | | | |
| 9. Relatedness | r | .224 | .229 | .227 | .140 | .111 | .368* | .167 | .470*** | 1 | | |
| | N | 30 | 30 | 30 | 30 | 30 | 41 | 42 | 42 | 42 | | |
| 10. PA Liking | r | .070 | .053 | -.015 | .108 | .106 | .312** | .380* | .124 | .045 | 1 | |
| | N | 83 | 83 | 83 | 83 | 83 | 103 | 42 | 42 | 42 | 108 | |
| 11. PA Importance | r | .178 | .026 | .167 | .065 | .054 | .142 | .072 | .452** | -.123 | .205* | 1 |
| | N | 84 | 84 | 84 | 84 | 84 | 104 | 42 | 42 | 42 | 108 | 109 |

**Table 6**

| Treatment-6-Months | | 1 | 2 | 3 | 4 | 5 | 6 | 7 | 8 | 9 | 10 | 11 |
|---|---|---|---|---|---|---|---|---|---|---|---|---|
| 1. Parent Support | r | 1 | | | | | | | | | | |
| | N | 109 | | | | | | | | | | |
| 2. Logistic Support | r | .780$^{***}$ | 1 | | | | | | | | | |
| | N | 109 | 109 | | | | | | | | | |
| 3. Modeling | r | .793$^{***}$ | .594$^{***}$ | 1 | | | | | | | | |
| | N | 109 | 109 | 109 | | | | | | | | |
| 4. Communication | r | .518$^{***}$ | .407$^{***}$ | .599$^{***}$ | 1 | | | | | | | |
| | N | 109 | 109 | 109 | 109 | | | | | | | |
| 5. Restricting SA | r | .786$^{***}$ | .357$^{***}$ | .358$^{***}$ | .263$^{**}$ | 1 | | | | | | |
| | N | 109 | 109 | 109 | 109 | 109 | | | | | | |
| 6. Pet Support | r | -.164 | -.209 | -.090 | .002 | -.114 | 1 | | | | | |
| | N | 63 | 63 | 63 | 63 | 63 | 84 | | | | | |
| 7. Autonomy | r | .168 | .306 | -.010 | .157 | .137 | .264 | 1 | | | | |
| | N | 25 | 25 | 25 | 25 | 25 | 33 | 34 | | | | |
| 8. Competence | r | .212 | .153 | .162 | .123 | .205 | .183 | .545$^{***}$ | 1 | | | |
| | N | 25 | 25 | 25 | 25 | 25 | 33 | 34 | 34 | | | |
| 9. Relatedness | r | .228 | .289 | .189 | .265 | .129 | .110 | .381$^{*}$ | .610$^{***}$ | 1 | | |
| | N | 25 | 25 | 25 | 25 | 25 | 33 | 34 | 34 | 34 | | |
| 10. PA Liking | r | -.019 | -.038 | -.103 | -.087 | .067 | .206 | .331 | .577$^{**}$ | .323 | 1 | |
| | N | 68 | 68 | 68 | 68 | 68 | 84 | 34 | 34 | 34 | 89 | |
| 11. PA Importance | r | -.216 | -.213 | .285$^{*}$ | -.176 | -.061 | .278$^{*}$ | .295 | .270$^{**}$ | .146 | .273$^{**}$ | 1 |
| | N | 68 | 68 | 68 | 68 | 68 | 84 | 34 | 34 | 34 | 89 | 89 |

## Appendix B: Correlation between Pet Support and Physical Activity Attitude between Boys and Girls

To explore the extent to which the relationship between perceived pet support and children's physical activity attitudes (i.e., PA liking and PA importance) differed between girls and boys in the treatment condition, a series of post hoc correlations were calculated. For girls, pet support was correlated with PA liking at $r = .20$, $p = .21$ at three months and $r = .30$, $p = .07$ at six months. Pet support was correlated with PA importance at $r = .29$, $p = .06$ at 3 months and $r = .56$, $p < .001$ at 6 months. For boys, pet support was correlated with PA liking at $r = .37$, $p = .004$ at three months and $r = .17$, $p = .25$ at six months. Pet support was correlated with PA

importance at $r$ =.08, $p$ = .56 at 3 months and $r$ = .13, $p$ = .40 at 6 months. With the expectation of PA liking at 3 months, the general trend suggests that pet support has a stronger effect on girls' PA attitudes than boys' attitudes. Thus, these analyses provide some preliminary evidence that the virtual agent used in this study (i.e., a virtual pet) was most effective at fostering a positive view of exercise for girls.
